# Supplementary material for: The lhfpl5 Ohnologs lhfpl5a and lhfpl5b Are Required for Mechanotransduction in Distinct Populations of Sensory Hair Cells in Zebrafish
Source: Front Mol Neurosci. 2020 Jan 15;12:320. doi: 10.3389/fnmol.2019.00320 (PMC6974483; doi:10.3389/fnmol.2019.00320)
Supplement: Supplementary file 1 [file Data_Sheet_1.pdf]

Supplementary Table 1. Lhfpl5 proteins used to construct the phylogenetic tree in Figure 1A.

Common name	Species	Phylogenetic Order	Gene name	Accession number
Seahorse	*Hippocampus comes*	Syngnathiformes	*lhfpl5a*	XP_019736131.1
			lhfpl5b	XP_019716386.1
Salmon (Atlantic)	*Salmo salar*	Salmoniformes	*lhfpl5a*	XP_014021466.1
			lhfpl5b	XP_014002848.1
Pufferfish	*Takifugu rubripes*	Tetraodontiformes	*lhfpl5a*	ENSTRUP00000020283.2
			lhfpl5b	ENSTRUP00000041734.2
Platyfish	*Xiphophorus maculatus*	Cyprinodontiformes	*lhfpl5a*	ENSXMAP00000020967.1
			lhfpl5b	ENSXMAP00000010306.1
Medaka	*Oryzias latipes*	Beloniformes	*lhfpl5a*	ENSORLP00000042616.1
			lhfpl5b	ENSORLP00000008018.2
Zebrafish	*Danio rerio*	Cypriniformes	*lhfpl5a*	ENSDARP00000066188.3
			lhfpl5b	ENSDARP00000073423.4
Cavefish (Blind)	*Astyanax mexicanus*	Characiformes	*lhfpl5a*	ENSAMXP00000035004.1
			lhfpl5b	ENSAMXP00000007271.2
Stickleback (3-spine)	*Gasterosteus aculeatus*	Gasterosteiformes	*lhfpl5a*	ENSGACP00000010149.1
			lhfpl5b	ENSGACP00000010766.1
Tilapia	*Oreochromis niloticus*	Cichliformes	*lhfpl5a*	ENSONIP00000024352.1
			lhfpl5b	ENSONIP00000014897.1
Perch (Climbing)	*Anabas testudineus*	Perciformes	*lhfpl5a*	ENSATEP00000035217.1
			lhfpl5b	ENSATEP00000029406.1
Eel (Zig-zag)	*Mastacembelus armatus*	Synbranchiformes	*lhfpl5a*	ENSMAMP00000013383.1
			lhfpl5b	ENSMAMP00000016042.1
Cod	*Gadus morhua*	Gadiformes	*lhfpl5a*	ENSGMOP00000012109.1
			lhfpl5b	ENSGMOP00000016613.1
Pike (Northern)	*Esox lucius*	Esociformes	*lhfpl5a*	ENSELUP00000020567.1
			lhfpl5b	ENSELUP00000014071.1
Spotted gar	*Lepisosteus oculatus*	Lepisosteiformes	*lhfpl5*	ENSLOCP00000013703.1
Xenopus	*Xenopus tropicalis*	Anura	*lhfpl5*	ENSXETP00000058779.1
Chicken	*Gallus gallus*	Galliformes	*lhfpl5*	ENSGALP00000053562.1
Mouse	*Mus musculus*	Rodentia	*lhfpl5*	ENSMUSP00000156557.1
Human	*Homo sapiens*	Primates	*lhfpl5*	ENSP00000493955.1

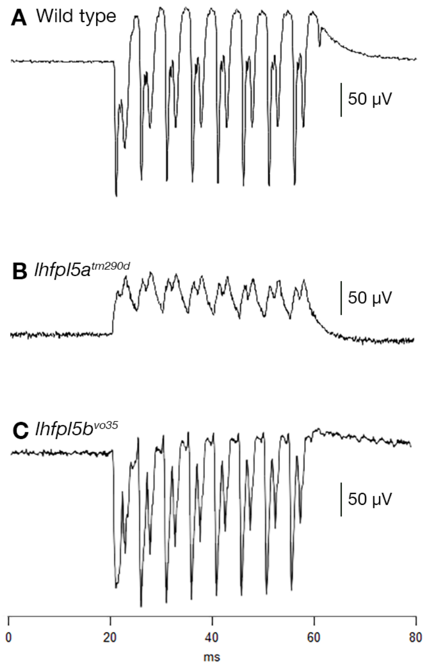

D

Genotype	n	Mean	Median	Std. Dev.	25-75 pctl.
Siblings (*tm290d*)	5	296.20	213.02	121.76	211.57 - 399.02
lhfpl5a^{tm290d}	7	53.06	51.14	10.48	48.83 - 59.05
Siblings (*vo35*)	4	243.84	233.35	96.74	190.78 - 286.41
lhfpl5b^{vo35}	6	280.86	185.64	196.47	166.67 - 301.01

Supplementary Figure 1. Representative microphonic traces from the inner ears of 3 day-old wild type (**A**), *lhfpl5a*^{tm290d} (**B**), and *lhfpl5b*^{vo35} (**C**) larvae. **D** – Table of the n-values, mean, median, standard deviation, and 25 - 75 percentile values for the first peak microphonic values graphed in Figure 3A.

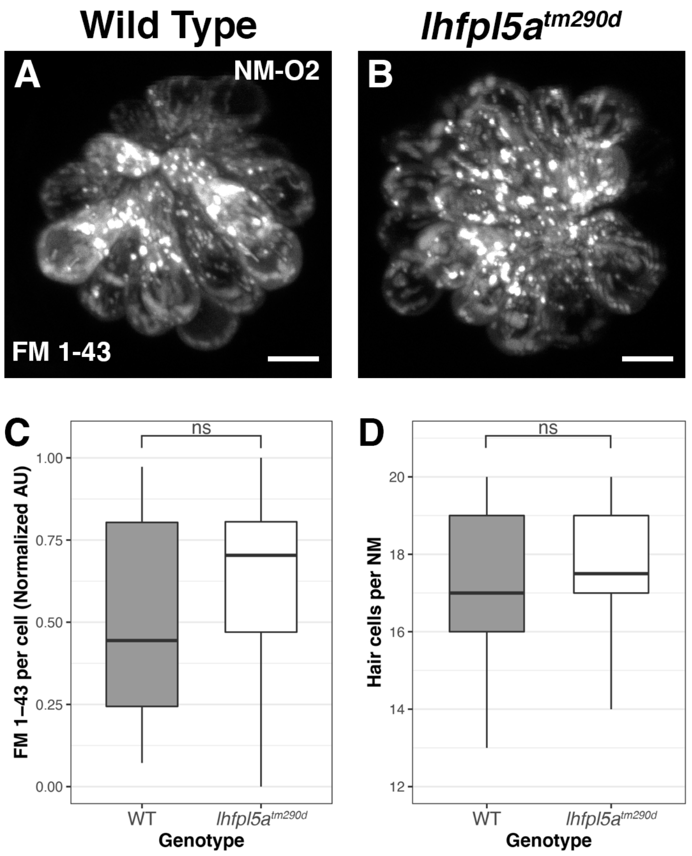

Supplementary Figure 2. Comparison of basal MET channel activity in neuromasts from wild type and *lhfpl5a^{tm290d}* mutant larvae. (**A, B**) Representative images of FM 1-43 fluorescence in lateral line neuromasts from wild type (A) and *lhfpl5a^{tm290d}* (B) mutants at 5 dpf. (**C**) Quantification of normalized FM 1-43 fluorescence intensity per hair cell in 5 dpf neuromasts (n = 6 WT, 6 *lhfpl5a^{tm290d}* larvae, 3 NMs per larvae). The box plots cover the inter-quartile range (IQR), and the whiskers represent the minimum and maximum datapoints within 1.5 times the IQR. p = 0.2229; ns = not significant. (**D**) Quantification of hair cell number in neuromasts from 5 dpf *lhfpl5a^{tm290d}* mutants and wild-type siblings, as determined by counting FM-positive hair cells. The same larvae and neuromasts were used as in C. p = 0.5828; ns = not significant by Welch's t-test.

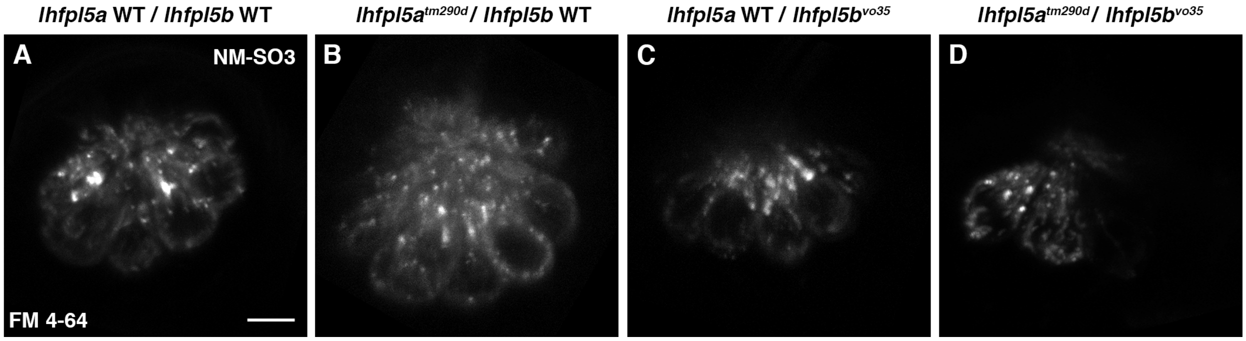

Supplementary Figure 3. Examples of residual basal MET channel activity in the SO3 neuromast hair cells of *lhfpl5b*^{vo35} mutants and *lhfpl5a*^{tm290d}; *lhfpl5b*^{vo35} double mutants. FM 4-64 labeled SO3 neuromasts from wild type (**A**), *lhfpl5a*^{tm290d} (**B**), *lhfpl5b*^{vo35} (**C**), and *lhfpl5a*^{tm290d}; *lhfpl5b*^{vo35} (**D**) larvae at 5 dpf. Scale bar = 5 µm, applies to all panels.

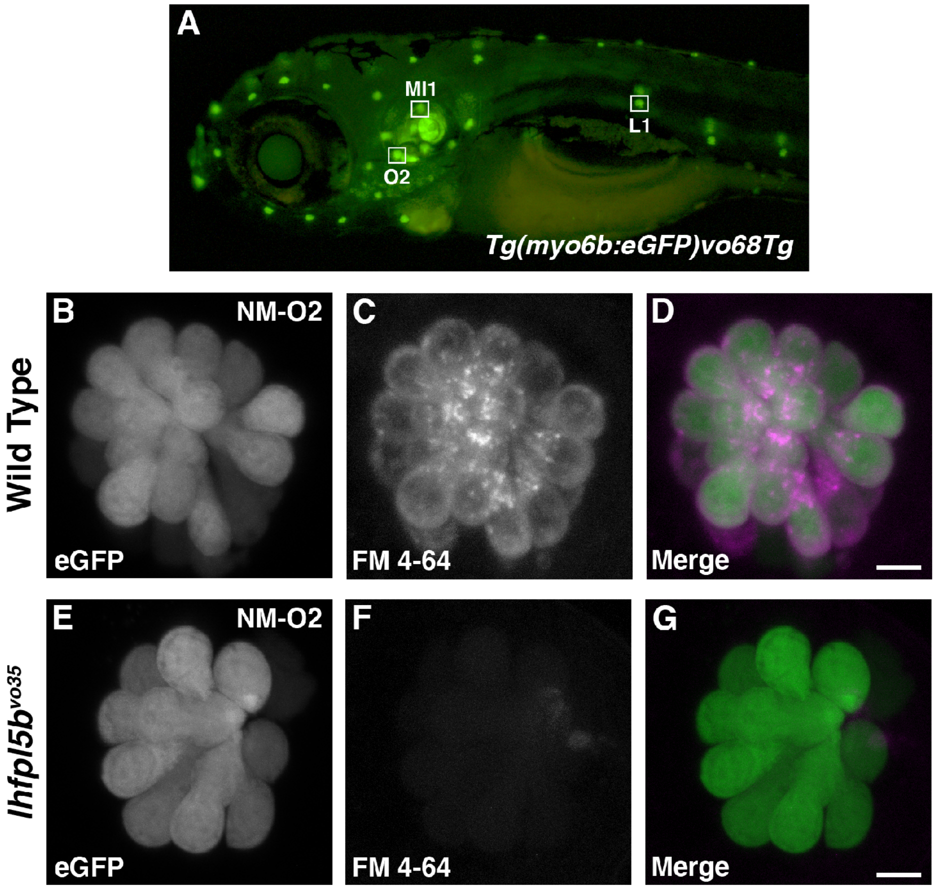

Supplementary Figure 4. (**A**) *Tg(myo6b:GFP)vo68Tg* larvae at 5 dpf. The neuromasts used for hair cell counting are labeled. (**B – G**) Representative images of GFP and FM 4-64-labeled hair cells in 5 dpf wild type and *lhfpl5b*^{vo35} mutant *Tg(myo6b:GFP)vo68Tg* larvae. These images are from larvae that make up part of the data set quantified in Figure 4K. Scale bar = 5 µm.

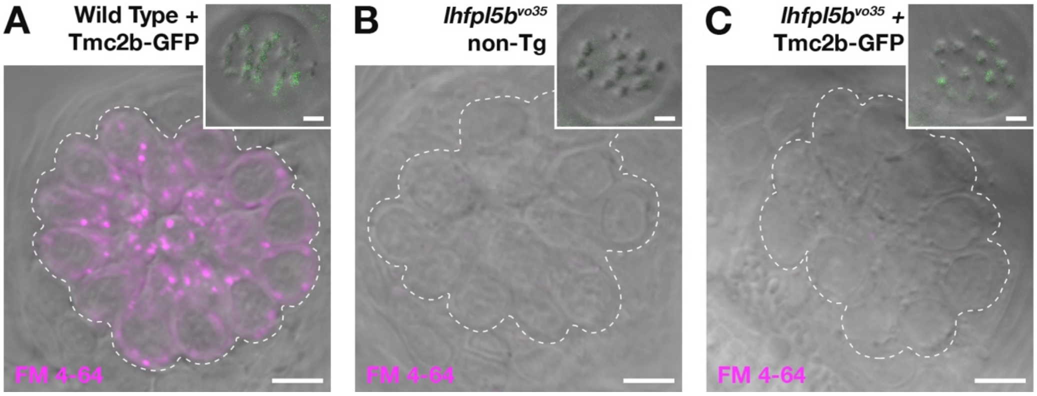

Supplementary Figure 5. Bundle-localized Tmc2b-GFP does not restore basal MET channel activity to neuromast hair cells of *lhfpl5b*^{vo35} mutants. (**A-C**) Representative images of neuromasts labeled with FM 4-64 from wild type Tmc2b-GFP *vo28Tg* (**A**), non-transgenic *lhfpl5b*^{vo35} (**B**), and *vo28Tg*; *lhfpl5b*^{vo35} (**C**) larvae at 7 dpf. Dashed lines outline the cluster of hair cells in each neuromast. Insets show the neuromast hair bundles from the same neuromast in the main panel. Scale bars = 5 µm in A-C; 2 µm for the bundle insets.

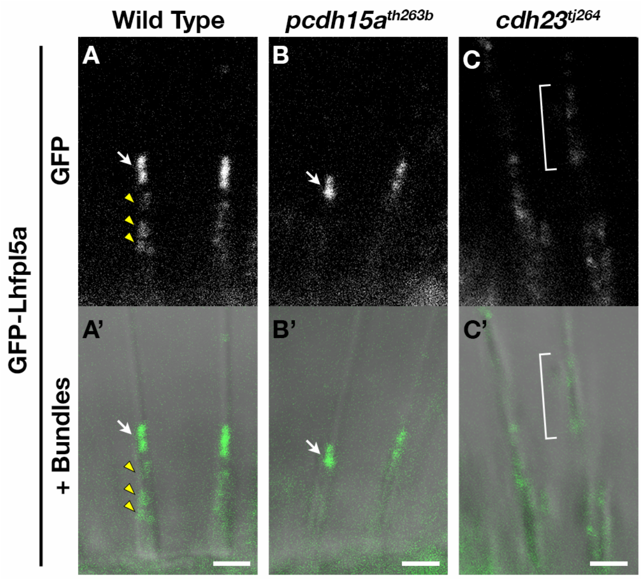

Supplementary Figure 6. Mislocalization of GFP-Lhfpl5a in different mutant alleles of *pcdh15a* and *cdh23* as shown in Figure 6. Representative images of GFP-Lhfpl5a (*vo23Tg*) in the lateral cristae hair bundles of wild type (**A, A'**) and $pcdh15a^{th263b}$ (**B, B'**), and $cdh23^{tj264}$ (**C, C'**) mutants. The GFP-only channel is shown in panels A - C and overlaid with a light image of the bundles in A' - C'. White arrows indicate GFP signal in the presumptive kinocilial linkages, yellow arrow heads indicate GFP signal in the stereocilia, and brackets indicate GFP signal in the kinocilium. Scale bars = 2 µm in A-C'.
